# Observer‐oriented approach improves species distribution models from citizen science data

**DOI:** 10.1002/ece3.6832

**Published:** 2020-09-26

**Authors:** Pietro Milanesi, Emiliano Mori, Mattia Menchetti

**Affiliations:** ^1^ Swiss Ornithological Institute Sempach Switzerland; ^2^ Istituto di Ricerca sugli Ecosistemi Terrestri Consiglio Nazionale delle Ricerche Sesto Fiorentino Firenze Italy; ^3^ Department of Biology University of Florence Sesto Fiorentino Florence Italy; ^4^ Institut de Biologia Evolutiva CSIC‐Universitat Pompeu Fabra Barcelona Spain

**Keywords:** biodiversity platforms, ecological niche modeling, mammals, sampling effort, selection of pseudo‐absences, spatial ecology

## Abstract

Citizen science platforms are increasingly growing, and, storing a huge amount of data on species locations, they provide researchers with essential information to develop sound strategies for species conservation. However, the lack of information on surveyed sites (i.e., where the observers did not record the target species) and sampling effort (e.g., the number of surveys at a given site, by how many observers, and for how much time) strongly limit the use of citizen science data. Thus, we examined the advantage of using an observer‐oriented approach (i.e., considering occurrences of species other than the target species collected by the observers of the target species as pseudo‐absences and additional predictors relative to the total number of observations, observers, and days in which locations were collected in a given sampling unit, as proxies of sampling effort) to develop species distribution models. Specifically, we considered 15 mammal species occurring in Italy and compared the predictive accuracy of the ensemble predictions of nine species distribution models carried out considering random pseudo‐absences versus observer‐oriented approach. Through cross‐validations, we found that the observer‐oriented approach improved species distribution models, providing a higher predictive accuracy than random pseudo‐absences. Our results showed that species distribution modeling developed using pseudo‐absences derived citizen science data outperform those carried out using random pseudo‐absences and thus improve the capacity of species distribution models to accurately predict the geographic range of species when deriving robust surrogate of sampling effort.

## Graphical abstract

We examined the advantages of using data available on Citizen science open‐access platforms through an observer‐oriented approach (i.e., considering occurrences of species other than the target species collected by the observers of the target species as pseudo‐absences and additional predictors relative to the total number of observations, observers, and days in which locations were collected in a given sampling unit, as proxies of sampling effort) to develop species distribution models.

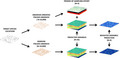

## 1 INTRODUCTION

Monitoring biodiversity is fundamental for conservation and sustainable use of natural resources but governmental, non‐governmental organizations (NGOs), and scientific agencies often lack financial resources to support long‐term biodiversity assessment by professional scientists and volunteers (Bland et al., 2015; Kelling et al., 2018). Collection of field‐data is often very expensive and requires a high economic and time effort, even to get a low amount of information, especially under the ongoing global economic crisis which led scientists to adapt to a period of limited availability of research funds (Cagnacci et al., 2012).

In this context, citizen science represents a powerful cost‐effective strategy to collect baseline scientific data by engaging common, that is, non‐professional, people, leveraging the growing public “environmental awareness” and the increase worldwide in wildlife enthusiasts (e.g., McCafferty, 2016; Silvertown et al., 2011; Willemen et al., 2015). Citizen science is becoming more and more popular as well as available online; actually, many organizations developed citizen science projects recruiting the wider public to provide large quantities of unstructured biodiversity data across large spatial and temporal extents (Amano et al., 2016; Danielsen et al., 2014; Mori & Menchetti, 2014; Pimm et al., 2014; Sullivan et al., 2014). Over 500 citizen science projects have been detected worldwide, through a systematic online research in 2017 (Pocock et al., 2017), promoted also by the widespread use of smartphones and tablets (Liebenberg et al., 2017; Wang et al., 2014) which have greatly simplified the procedure to upload records on online platforms (Pocock et al., 2017). Monitoring biodiversity through citizen science projects is having a great influence in ecology (Dickinson et al., 2010) and a big variety of platforms are running nowadays (e.g., iNaturalist.org, essentially about collating casual observations, and eBird.org, strongly encouraging complete lists with associated effort while also allowing for less structured recordings). Citizen science data often result in a high number of occurrences recorded over large areas (i.e., countries or continents), and time spans and at relatively low costs (Hobson et al., 2017; Mori, et al., 2017; Paul et al., 2014; Willemen et al., 2015). Opportunistic citizen data have been shown to provide researchers with well‐approximated distribution ranges (or with further data on existing occurrences) and predictions of habitat use, necessary to address functional conservation efforts (e.g., Bruce et al., 2014; Tye et al., 2016). Moreover, citizen science data on online platforms has allowed researchers to perform studies on biogeography, alien species range expansion, species natural history, and interspecific interactions (Chandler et al., 2017; Menchetti et al., 2019; Mori et al., 2018; Mori & Menchetti, 2014; Sullivan et al., 2014; Vendetti et al., 2018). Therefore, citizen science is playing an important role in improving conservation biology, including also natural resource management and environmental preservation (Devictor et al., 2010; McKinley et al., 2017; Van der Wal et al., 2015).

Citizen science has the potential to remarkably increase our biodiversity knowledge (Pimm et al., 2014), but it can be challenging to identify citizen data that effectively monitor biodiversity (Kelling et al., 2018). Specifically, the use of citizen science data for biodiversity assessment is limited by several concerning factors including the lack of absence data and information on sampling effort (Crall et al., 2011, 2015; Dickinson et al., 2010; Kamp et al., 2019; Kelling et al., 2018), leading to limited interpretations (Ottinger, 2010; Conrad & Hilchey, 2011). These are serious issues which may strongly influence the accuracy of species distribution models (SDMs). SDMs combine species presence/absence locations with a set of environmental covariates (e.g., climatic variables) to identify factors related to species occurrence and thus predict species distribution to unsampled sites across a landscape (Elith & Leathwick, 2009). Ideally, species locations should be randomly distributed through the environmental space and sampling effort equal across the landscape, which is rarely the case citizen science data (Yackulic et al., 2013). When developing SDMs, the lack of absence data, and/or information on sampling effort can both inflate the species' presence in localized areas and cause some environmental habitats to be overlooked, increasing the likelihood of type I errors (false positives) and thus generating misleading predictions (Roy‐Dufresne et al., 2019). To overcome these issues, presence‐only SDMs use pseudo‐absences instead of real absences to predict species distribution but there is still no consensus on the best way to sample these pseudo‐absences (Barbet‐Massin et al., 2012).

Surprisingly, most of the studies using citizen science data to develop SDMs do not attempt to provide reliable pseudo‐absences data but rather investigate data quality developing protocols tested on citizen science (Delaney et al., 2008; Genet & Sargent, 2003), as well as smart filters to flag doubtful data uploaded on online databases, often using information contained within the citizen data, for example, observation date, ID of the observer (Crall et al., 2015). However, while data from online portals are not without limitations, data stored in citizen science projects that collect sufficient contextual information describing the observation process can be used to generate increasingly accurate information about the distribution and abundance of organisms through SDMs (Elith & Leathwick, 2009; Kelling et al., 2018).

Thus, in this study, we tested a new approach, namely “observer‐oriented” approach, to improve SDMs, identifying reliable pseudo‐absences as well as accounting for (pseudo‐) sampling effort using citizen science data collected by the same observers of the target species. Basically, instead of using random pseudo‐absences, our approach consists of using records of species of other than the target species collected by the observers of the target species as pseudo‐absences and adding proxies of sampling effort (i.e., the number of total observations, observers, and days in which locations were collected in a given sampling unit) as additional predictors in SDMs. We assumed that (a) a given observer of a given species would collect locations of such species when they will find it in the field and that (b) essential information available in online citizen science repositories could be used to derive reliable proxies of sampling effort.

Thus, our aim is to test if SDMs based on “observer‐oriented” approach outperform (i.e., result in higher predictive accuracy than) those develop using random pseudo‐absences.

## 2 MATERIALS AND METHODS

### 2.1 Presences and observer‐oriented pseudo‐absences

We considered presence locations of 15 terrestrial mammal species (Table 1) collected by citizen scientists during the period 2010–2018 in Italy, extracted from the iNaturalist project “Mammiferi d’Italia” (www.inaturalist.org/projects/mammiferi‐d‐italia) which gathers all the observations of Italian mammals uploaded in the platform and where species identification is supervised by the authors EM and MM. We considered only species locations for which geographic coordinates were provided. The citizen science website iNaturalist is an open‐access and open‐source platform aimed to record biodiversity worldwide. This platform allows downloading all the occurrences using specific queries (i.e., taxon, place, user/observer, date, etc.).

**Table 1:** Number of presence occurrences, their observers and resulting total pseudo‐absences collected for the 15 species of terrestrial mammals considered in this study between 2010 and 2018

Species	Occurrences	Observers	Observer‐oriented pseudo‐absences
*Capreolus capreolus*	976	232	22,116
*Vulpes vulpes*	731	245	22,299
*Myocastor coypus*	673	280	21,892
*Rupicapra rupicapra*	610	141	15,889
*Erinaceus europaeus* ^a^	577	247	20,381
*Sciurus vulgaris*	536	233	24,290
*Sus scrofa*	475	151	18,557
*Meles meles*	439	154	20,346
*Lepus europaeus*	399	159	20,207
*Sylvilagus floridanus*	301	108	15,862
*Canis lupus*	284	73	12,374
*Cervus elaphus*	270	100	14,354
*Hystrix cristata*	193	88	14,795
*Sciurus carolinensis*	141	83	13,549
*Dama dama*	96	52	11,055

^a^ We considered only data collected between April and October to avoid false pseudo‐absences due to species hibernation.

To select pseudo‐absences of each considered species, we listed their relative observers and then extracted, from iNaturalist online platform, all the locations of all the species (i.e., including both plants and animals) collected by these observers. Similar to presence locations of our 15 target species, we considered only data collected during the period 2010–2018 in Italy for which geographic coordinates were provided.

### 2.2 Study area

Our study area corresponds to the whole Italian territory (7°49′–13°91′ E; 45°–42° 39′ N), which is about 300,000 km^2^, ranging from 0 to 4,810 m a. s. l. with a climatic gradient from temperate to continental, to alpine, resulting in high habitat diversity. The ongoing human population abandonment in the hilly and mountainous parts of our study area started already 50–60 years ago, lead to a dramatic decrease of agriculture in favor of shrub‐lands, woods, and forests. Forests, composed by broadleaf or mixed woods and, to a lesser extent, by coniferous forests are mainly located on the Alps and the Apennines. Here, grasslands are mainly used only for livestock grazing. Thus, the environment results in a patchy landscape pattern of forests and open‐areas across large zones where most of the human population live in the main valleys, big cities along the coasts and plains.

### 2.3 Predictor variables

We initially collected 43 predictor variables contiguously available for the entire study area (Table S1). We considered three topographic variables (altitude, slope, and landscape roughness), derived from a digital elevation model of Italy with a spatial resolution of 20 m (www.sinanet.isprambiente.it), 19 bioclimatic predictors collected from the WorldClim dataset (www.worldclim.org/version2 at a spatial resolution of 30 arc‐second, ≈1 km), 11 land cover variables (percentage of coniferous, deciduous, and mixed forests, distance to forests, croplands, grasslands, shrub‐lands, water courses, distance to water courses, rocky areas, and habitat diversity) derived from CORINE Land Cover vector data (European Environment Agency 2012; www.sinanet.isprambiente.it). Moreover, we also included four forest structure variables namely density of trees (at a spatial resolution of 1 km; www.elischolar.library.yale.edu/yale_fes_data/1/; www.figshare.com/articles/Global_map_of_tree_density/3179986), wood biomass (1 km resolution; www.wageningenur.nl/grsbiomass), canopy height (at a spatial resolution of 1 km; www.landscape.jpl.nasa.gov/), and canopy height roughness (as a measure of variation in canopy height, a proxy for the heterogeneity of the vegetation; Froidevaux et al., 2016).

Finally, we also considered six anthropogenic features: the percentage and distance to human settlements (i.e., urban areas and villages also derived from the CORINE Land Cover 2012), density of and distance to roads (OpenStreetMap; www.openstreetmap.org), human population density (GEOSTAT 2011 1 × 1 km grid dataset – Eurostat – European Commission;

www.ec.europa.eu/eurostat/web/gisco/geodata/reference‐data/population‐distribution‐demography; Table S1) and artificial night‐time light brightness (NOAA, NPP VIIRS – NASA 2012 with a spatial resolution of 350 m; www.ngdc.noaa.gov/eog/viirs/download_dnb_composites.html).

All predictor variables were resampled at a 1 × 1 km grid cell size, and we calculated the Variance Inflation Factor (VIF; Zuur et al., 2010) to avoid that multicollinearity among predictors negatively affected SDMs. Specifically, we used a stepwise variable selection procedure in which variables were removed till the highest VIF value was <3 (Zuur et al., 2010). Thus, we removed 17 predictors because of VIF > 3 (highly related to other predictors; Zuur et al., 2010; Table S1).

### 2.4 Species distribution models

Similar to Milanesi et al. (2019), to develop SDMs avoiding biased estimation due to single model uncertainty (Thuiller et al., 2009), we calculated the weighted ensemble prediction (wEP, weighted by the true skills statistic, TSS; see below) averaging nine different SDMs namely (a) artificial neural networks (ANN; Ripley, 2007), (b) boosted regression trees (BRT; Friedman, 2001), (c) flexible discriminant analyses (FDA; Hastie et al., 1994), (d) generalized additive models (GAM; Hastie & Tibshirani, 1990), (e) generalized linear models (GLM; McCullagh & Nelder, 1989), (f) multivariate adaptive regression splines (MARS; Friedman, 1991), (g) maximum entropy algorithm (MAXENT; Phillips et al., 2006), (h) MAXENT model using the glmnet package (Friedman et al., 2010) for regularized generalized linear models (MAXNET; Phillips et al., 2017) and (i) random forests (RF; Breiman, 2001). We developed SDMs through the packages BIOMOD2 (Thuiller et al., 2016) and MAXNET (Phillips et al., 2017) in R (R Core Team, 2013).

We found evidence of spatial autocorrelation among models’ residuals through Moran's *I* correlogram, and thus, similarly to Pasinelli et al. (2016), we included *x*‐ and *y*‐coordinates of species locations and their interaction in SDMs (then, models residuals where no longer spatially autocorrelated).

### 2.5 *Comparison of SDMs developed using random* versus*. observer‐oriented pseudo‐absences*

**Figure 1:**
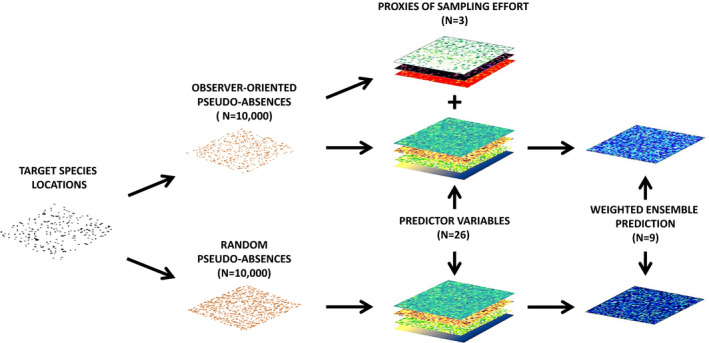
Conceptual framework showing the steps followed to develop species distribution models based on “observer‐oriented” approach (first and second line) and random pseudo‐absences (third line)

We develop two sets of SDMs, alternatively using (a) totally random pseudo‐absences (hereafter rpa‐SDMs) and (b) observer‐oriented approach (hereafter ooa‐SDMs, i.e., considering other than target species locations collected by the observers of the target species as pseudo‐absences and additional predictors related to the total number of observations, observers and days in which locations were collected in a given sampling unit, as proxies and to account for sampling effort; Figure 1).

To avoid the possibility that different sample sizes of observer‐oriented pseudo‐absences (Table 1) might bias our results, we randomly selected a total of 10,000 observer‐oriented pseudo‐absences for ooa‐SDMs (equal to the number of random pseudo‐absences in rpa‐SDMs; we repeated this procedure 10 times and found consistent results of the further analyses).

By using a random subsample of 90% of the locations to calibrate the models and the remnant 10% to evaluate them (Thuiller et al., 2009), we carried out 10‐fold cross‐validations to test the predictive accuracy of both rpa‐ (considering random pseudo‐absences) and ooa(observer‐oriented pseudo‐absences)‐SDMs. Specifically, we considered two widely used indices to evaluate model performance: (a) the area under the receiver operating characteristic curve (AUC) and (b) the true skills statistic (TSS). AUC ranges between 0 and 1 (worse than a random model and best discriminating model, respectively) while TSS between −1 and 1 (higher values indicate a good predictive accuracy, while 0 indicates random prediction). For a visual comparison, we rescaled the resulting maps derived by rpa‐ and ooa‐SDMs to range between 0 and 1. Values close to 0 indicate low suitability while close to 1 indicate high suitability.

## 3 RESULTS

We considered a total of 6,701 occurrences of our target species (Figure 2), ranging from 96 for the fallow deer *Dama dama* to 976 for the roe deer *Capreolus capreolus*. All these locations were collected from a total of 957 observers, ranging from 52 for the fallow deer to 280 for the coypu *Myocastor coypus*, who collected a total of 237,010 non‐target species occurrences (Figure 2), ranging from 11,055 for the fallow deer to 24,290 for the red squirrel *Sciurus vulgaris*, which we initially considered as observer‐oriented pseudo‐absences (Table 1; Figs. S1–S15).

**Figure 2:**
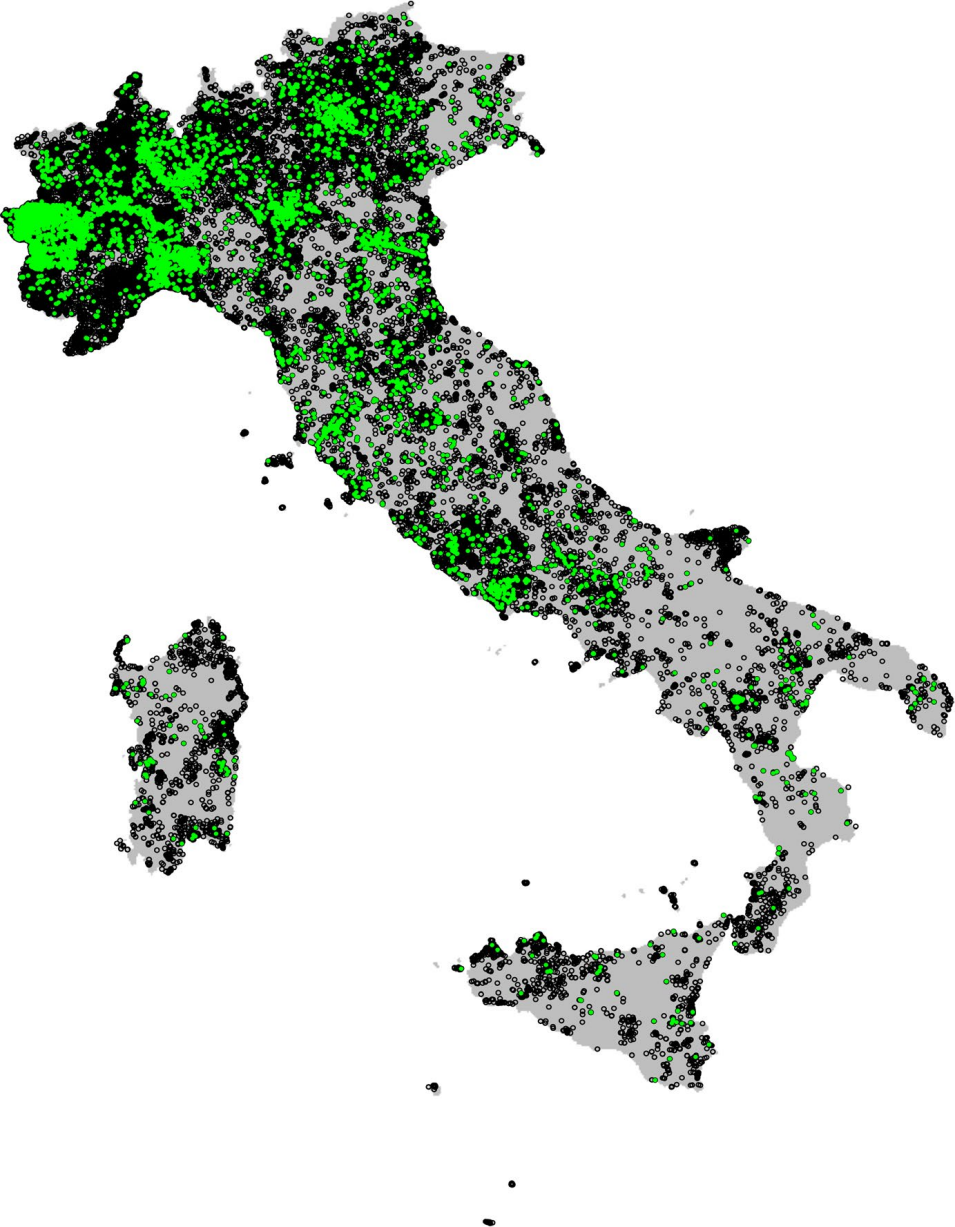
Study area (in gray). Target species locations in green, total observer‐oriented pseudo‐absences (i.e., considering other than target species locations collected by the observers of the target species) in black

**Figure 3:**
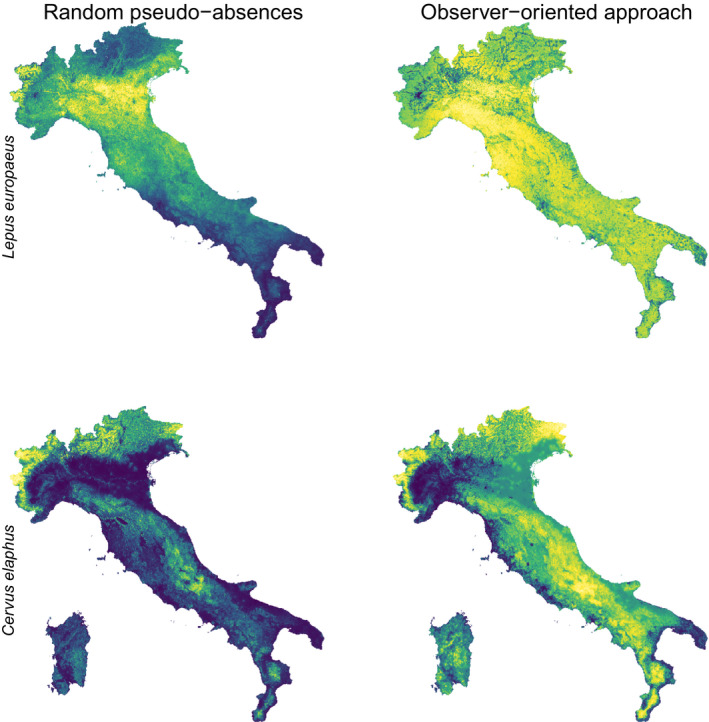
Example of resulting weighted ensemble predictions for the European brown hare (first line) and the red deer (second line) derived from nine different species distribution models carried out alternatively using random pseudo‐absences (left) and “observer‐oriented” approach (right). Blue‐yellow scale indicates low‐high suitability

We generally found that ooa‐SDMs had higher predictive accuracy than rpa‐SDMs, considering both AUC and TSS. Specifically, the red fox *Vulpes vulpes* and the gray squirrel *Sciurus carolinensis* showed the highest and the lowest validation statistics, respectively, for both AUC and TSS (Table 2). AUC values of rpa‐SDMs ranged from 0.639 to 0.906 while those of ooa‐SDMs ranged from 0.767 to 0.945, on the other side TSS values ranged from 0.271 to 0.776 of rpa‐SDMs, while those of ooa‐SDMs ranged from 0.436 to 0.814 (Table 2; Figure 3).

**Table 2:** Ten‐fold cross‐validations of the weighted ensemble prediction (wEP) of nine species distribution models carried out on 15 species of terrestrial mammals. Area Under the Curve (AUC) ranges between 0 and 1 (worse than random and best discriminating model, respectively) while True Skill Statistic (TSS) between −1 and 1 (high values indicate good predictive accuracy, 0 indicates random prediction). Average values ± standard deviations alternatively using 10,000 random or observer‐oriented pseudo‐absences are shown

Species	Random pseudo‐absences		Observer‐oriented approach	
	AUC	TSS	AUC	TSS
*Capreolus capreolus*	0.756 ± 0.026	0.433 ± 0.043	0.834 ± 0.026	0.546 ± 0.039
*Vulpes vulpes*	0.639 ± 0.048	0.271 ± 0.079	0.767 ± 0.022	0.436 ± 0.044
*Myocastor coypus*	0.796 ± 0.032	0.494 ± 0.063	0.858 ± 0.024	0.568 ± 0.052
*Rupicapra rupicapra*	0.905 ± 0.026	0.691 ± 0.066	0.914 ± 0.017	0.693 ± 0.045
*Erinaceus europaeus*	0.784 ± 0.027	0.504 ± 0.047	0.852 ± 0.026	0.572 ± 0.051
*Sciurus vulgaris*	0.705 ± 0.054	0.351 ± 0.075	0.825 ± 0.019	0.513 ± 0.031
*Sus scrofa*	0.697 ± 0.021	0.348 ± 0.031	0.824 ± 0.024	0.532 ± 0.053
*Meles meles*	0.685 ± 0.053	0.344 ± 0.083	0.799 ± 0.031	0.477 ± 0.048
*Lepus europaeus*	0.751 ± 0.033	0.424 ± 0.053	0.822 ± 0.041	0.541 ± 0.076
*Sylvilagus floridanus*	0.808 ± 0.043	0.545 ± 0.073	0.874 ± 0.022	0.616 ± 0.046
*Canis lupus*	0.747 ± 0.068	0.464 ± 0.104	0.835 ± 0.032	0.582 ± 0.079
*Cervus elaphus*	0.831 ± 0.053	0.589 ± 0.093	0.882 ± 0.049	0.676 ± 0.096
*Hystrix cristata*	0.724 ± 0.067	0.439 ± 0.091	0.774 ± 0.059	0.446 ± 0.071
*Sciurus carolinensis*	0.906 ± 0.021	0.776 ± 0.048	0.945 ± 0.028	0.814 ± 0.083
*Dama dama*	0.844 ± 0.077	0.621 ± 0.138	0.856 ± 0.074	0.691 ± 0.151

We recorded the highest difference between AUC and TSS values of rpa‐ and ooa‐SDMs for the red fox and the wild boar *Sus scrofa*, respectively, while the lowest differences for both validation statistics were recorded for the Northern chamois *Rupicapra rupicapra* (Table 3).

**Table 3:** Difference between average values of Area Under the Curve (AUC) and True Skill Statistic (TSS) estimated by weighted ensemble prediction of nine species distribution models carried out on 15 species of terrestrial mammals alternatively using random pseudo‐absences and observer‐oriented approach

Species	Δ AUC	Δ TSS
*Capreolus capreolus*	0.078	0.113
*Vulpes vulpes*	0.128	0.165
*Myocastor coypus*	0.062	0.074
*Rupicapra rupicapra*	0.009	0.002
*Erinaceus europaeus*	0.068	0.068
*Sciurus vulgaris*	0.12	0.162
*Sus scrofa*	0.127	0.184
*Meles meles*	0.114	0.133
*Lepus europaeus*	0.071	0.117
*Sylvilagus floridanus*	0.066	0.071
*Canis lupus*	0.088	0.118
*Cervus elaphus*	0.051	0.087
*Hystrix cristata*	0.05	0.007
*Sciurus carolinensis*	0.039	0.038
*Dama dama*	0.012	0.07

## 4 DISCUSSION

In this study we compared SDMs developed using species occurrences derived from citizen science data but alternatively using random or observer‐oriented occurrences as pseudo‐absences. We found that the “observer‐oriented” approach outperforms the widely used random pseudo‐absences approach, and thus, we provided a better framework showing how opportunistic citizen science data can be used to develop more accurate species distribution models.

### 4.1 Citizen science data and species distribution models

The use of citizen science data has been initially advocated to assess species distribution at large scale, where standardized sampling is often impracticable (Mori et al., 2019; Van Strien et al., 2013). However, this method has been recently criticized due to uncertainties associated with underlying sampling processes (Mair & Ruete, 2016). While only citizen science projects can gather sufficient quantities of species locations, these data are inherently noisy and heterogeneous (Kelling et al., 2015). Moreover, citizen science datasets available on online platforms do not provide information on all sampling sites (even those were target species where absent) or on sampling effort, both of which are fundamental to distinguish evidence of true absence of the target species from merely insufficient effort to detect it (Croft et al., 2019).

While these aspects strongly limit the use of citizen science data in developing SDMs, we believe that there is a huge amount of valuable information available in citizen science datasets that deserve much attention and critical rethinking. Recently, researchers successfully explored the benefits of using citizen science data in combination with standardized data collected by professional field workers to estimate species distribution and abundance (Johnston et al., 2018; Kelling et al., 2020; Roy‐Dufresne et al., 2019; Tye et al., 2016). While these studies provided useful insights, in this research we considered only citizen science data to develop SDMs, and our results showed that citizen science data can be correctly used to develop SDMs with high predictive accuracy. Specifically, accounting for surrogates of sampling effort led to an overall increase in predictive accuracy as shown by the higher values of validation statistics of the SDMs carried out with observer‐oriented pseudo‐absences than those of SDMs carried out considering random pseudo‐absences. Thus, our results proved the usefulness of large citizen science datasets to estimate species distributions not only considering target species locations but also those of other species collected by the same observers of the target species as pseudo‐absences, accounting for the unequal sampling effort that could occur in site selection, in agreement with previous studies suggesting that records of other species may provide a suitable proxy to estimate survey effort (Phillips et al., 2009; Croft et al., 2019; van Strien et al., 2013). Thus, we believe that our “observer‐oriented” approach represents a new methodological way to develop more robust and accurate SDMs than those developed using random pseudo‐absences, potentially useful and widely applicable to many ecological contexts.

### 4.2 *Random* versus *observer‐oriented pseudo‐absences in SDMs*

Recently, Loy et al. (2019) revised the checklist of Italian mammals, with data over 120 species and their relative distributions, updated following the most recent scientific literature (cf. also Amori et al., 2008; Boitani et al., 2003). The checklist built by Loy et al. (2019) was totally based on an expert‐based approach (without considering data uploaded on iNaturalist) involving 21 top experts on Italian mammals. Considering this recent assessment, we generally found that the output maps of the observer‐oriented approach showed better approximations of distributions of all the selected mammalian species in this study, compared to those derived using random pseudo‐absences.

Specifically, the random models underestimated the actual distributions for many of our target species. For instance, widely distributed species, for example, the red deer *Cervus elaphus,* the fallow deer, the European brown hare *Lepus europaeus,* and the gray wolf *Canis lupus* showed a wider suitability in Northern regions, whereas being poorly represented in Southern ones, where citizen science records are few, suggesting that our observer‐oriented “pseudo‐absences” closely correspond to real species’ absences. Similarly, a gradient of decreasing suitability from Northern to Central and Southern regions in the resulting maps of SDMs carried out using random pseudo‐absences can be observed for the Eastern cottontail *Sylvilagus floridanus*, the Eurasian red squirrel, and the European roe deer. The alien Eastern gray squirrel shows invasive populations mainly in North‐Western Italy, but several nuclei also occur in North‐Eastern and Central Italy (Loy et al., 2019); the output maps of SDMs carried out using random pseudo‐absences highlighted only the main invaded areas, whereas the observer‐oriented clearly reflected also the occurrences of small and isolated populations (Di Febbraro et al., 2019).

On the other side, output maps carried out with the two different approaches provided reliable outputs for large and diurnal herbivores living in limited areas (e.g., the only Alpine area in Italy), such as the Northern chamois and the Alpine ibex *Capra ibex* (the latter was not included in this study). These species have precise habitat requirements and frequently attract citizen scientists and natural photographers (Brambilla et al., 2020; Mori, et al., 2018), suggesting that their true distribution would be well‐represented in citizen science platforms, i.e., species’ absences mainly correspond to where they have not been recorded and thus both random and “observer‐oriented” pseudo‐absences mainly correspond to absences. Similarly, also the distribution of the European hedgehog *Erinaceus europaeus* is well‐represented by both models. This small mammal is one of the most widespread mammal species in Italy (Loy et al., 2019), living in a number of habitat types ranging from woodland to urban areas (Amori et al., 2008).

Common mesomammals, for example, the red fox, the European badger *Meles meles*, the coypu, and the crested porcupine *Hystrix cristata*, frequently recorded as road‐kills, as well as the wild boar, consistently showed a medium‐high suitability throughout Italy, but at a lower level with respect to observer‐oriented models. This could be related to the fact that all those species are very widespread in Italy (Loy et al., 2019), and they could also be under‐recorded by citizen scientists. Biological characteristics of these species (e.g., nocturnal habits, elusiveness, particular habitat requirements, and scattered distribution) may lower their detectability, or citizen scientist may consider them as common and poorly important to be recorded.

## 5 CONCLUSIONS

Citizen science data could play a fundamental role in addressing challenges to biodiversity conservation, especially at broad scale. In many cases, they represent the only source of information but they are also likely to contain large biases (e.g., in sampling effort and spatial coverage; Dobson et al., 2020). In this study, we showed how accounting for such biases could improve model performance, providing accurate estimates of species distribution. Moreover, while the preparation and analysis of opportunistic data frequently requires a higher amount of money and effort than for more structured datasets (Cagnacci et al., 2012; Dobson et al., 2020), we argue that, thanks to the already existing R packages (i.e., “RINAT,””SPOCC”), it is relatively easy and straightforward to collect species occurrences from open‐access citizen science platforms such as iNaturalist. Furthermore, the crowdsource identification method on iNaturalist is also open to all worldwide experts and in the case of easily recognizable taxa does not require correction by the observer, thus making it suitable for revision at any time. This is relevant especially in light of ongoing national and continental atlas projects, for example, the Atlas of Italian Mammals (“*Atlante dei Mammiferi Italiani*”) and the second Atlas of European Mammals (EMMA II), respectively. This holds true also in light of the effect of climate and land use change on future species distribution (Della Rocca et al., 2019; Della Rocca & Milanesi, 2020; Milanesi et al., 2017; Mori et al., 2018).

Nevertheless, while providing more accurate estimates than standard SDMs (involving random pseudo‐absences), we stress that our approach represents a starting point on the development of SDMs totally based on presence‐only citizen science data. Unfortunately, due to the lack of data derived by structured surveys for our target species, we could not compare our results to those of comprehensive Atlas projects such as done in Johnston et al. (2018). Thus, we suggest that further studies should explore the inclusion of other parameters (e.g., observer’ skills, observation process) or even attempt to estimate abundance/density of the target species with citizen science data. In the meanwhile, promoting the adoption of standardized sampling schemes and spatial coverage will inevitably increase data quality and thus lead to even more robust results. Thus, we stress that a more structured approach to the collection of Citizen Science data is needed and should be encouraged wherever possible while making better use of existing presence‐only data as an interim measure.

## CONFLICT OF INTEREST

None declared.

## AUTHOR CONTRIBUTION

**Pietro Milanesi:** Conceptualization (lead); Data curation (lead); Formal analysis (lead); Investigation (lead); Methodology (lead); Resources (lead); Software (lead); Supervision (lead); Validation (equal); Writing‐original draft (lead); Writing‐review & editing (lead). **Emiliano Mori:** Conceptualization (supporting); Investigation (supporting); Resources (equal); Supervision (supporting); Writing‐original draft (equal); Writing‐review & editing (equal). **Mattia Menchetti:** Conceptualization (equal); Data curation (equal); Investigation (equal); Project administration (equal); Resources (equal).

## Supporting information

Appendix

## Data Availability

Species occurrences considered in this study are freely available at www.inaturalist.org/projects/mammiferi‐d‐italia, while GIS layers are freely available at www.sinanet.isprambiente.it, www.worldclim.org/version2, www.elischolar.library.yale.edu/yale_fes_data/1/, www.figshare.com/articles/Global_map_of_tree_density/3179986, www.wageningenur.nl/grsbiomass, www.landscape.jpl.nasa.gov/, www.openstreetmap.org, www.ec.europa.eu/eurostat/web/gisco/geodata/reference‐data/population‐distribution‐demography, www.ngdc.noaa.gov/eog/viirs/download_dnb_composites.html).
