# Supplementary material for: Observer‐oriented approach improves species distribution models from citizen science data
Source: Ecol Evol. 2020 Sep 26;10(21):12104–14. doi: 10.1002/ece3.6832 (PMC7663073; doi:10.1002/ece3.6832)

**APPENDIX**

Table S1. Predictor variables considered and their relative VIF (Variance Inflation Factor). Predictors with VIF>3 were not considered in developing species distribution models.

| **Predictor variable** | **Unit** | **VIF** |
| --- | --- | --- |
| Altitude | m a.s.l. | > 3 |
| Slope | ° | 1.844 |
| Landscape roughness | isoipses’ average length / cell side | > 3 |
| Bio 1 – Annual mean temperature | °C | > 3 |
| Bio 2 – Mean diurnal range | °C | > 3 |
| Bio 3 – Isothermality | °C x 100 | 1.618 |
| Bio 4 –Temperature seasonality | standard deviation *100 | > 3 |
| Bio 5 – Max temperature of warmest month | °C | > 3 |
| Bio 6 – Min temperature of coldest month | °C | > 3 |
| Bio 7 – Temperature annual range | °C | > 3 |
| Bio 8 – Mean temperature of wettest quarter | °C | 2.552 |
| Bio 9 – Mean temperature of driest quarter | °C | 1.775 |
| Bio 10 – Mean temperature of the warmest quarter | °C | > 3 |
| Bio 11 – Mean temperature of the coldest quarter | °C | > 3 |
| Bio 12 – Annual precipitation | mm | > 3 |
| Bio 13 – Precipitation of wettest month | mm | 1.857 |
| Bio 14 – Precipitation of driest month | mm | > 3 |
| Bio 15 – Precipitation seasonality | Coefficient of variation | 1.149 |
| Bio 16 – Precipitation of wettest quarter | mm | > 3 |
| Bio 17 – Precipitation of the driest quarter | mm | > 3 |
| Bio 18 – Precipitation of the warmest quarter | mm | > 3 |
| Bio 19 – Precipitation of the coldest quarter | mm | > 3 |
| Coniferous forests | % | 1.445 |
| Deciduous forests | % | 2.998 |
| Mixed forests | % | 1.507 |
| Distance to forests | m | 1.707 |
| Croplands | % | > 3 |
| Grasslands | % | 1.460 |
| Shrub-lands | % | 1.401 |
| Water courses | % | 1.078 |
| Distance to water courses | m | 1.154 |
| Rocky areas | % | 1.920 |
| Shannon’ habitat diversity index | *H’ = −Σ* ( *p_i_ × lnp_i_* ) | 1.596 |
| Density of trees | number/km^2^ | 2.075 |
| Wood biomass |  | 2.066 |
| Canopy height | m | 2.501 |
| Canopy height roughness | isoipses’ average length / cell side | 1.171 |
| Human settlements | % | 2.113 |
| Distance to human settlements | m | 1.896 |
| Density of roads | km/km^2^ | 1.790 |
| Distance to roads | m | 1.301 |
| Human population density | number/km^2^ | 1.618 |
| Artificial night-time light brightness | nw/cm^2^/sr | 2.245 |

Figure S1. Resulting weighted ensemble predictions for the grey wolf derived from nine different species distribution models carried out alternatively using random pseudo-absences (left) and observer-oriented’ approach (right). Blue-yellow scale indicates low-high probability of occurrence.


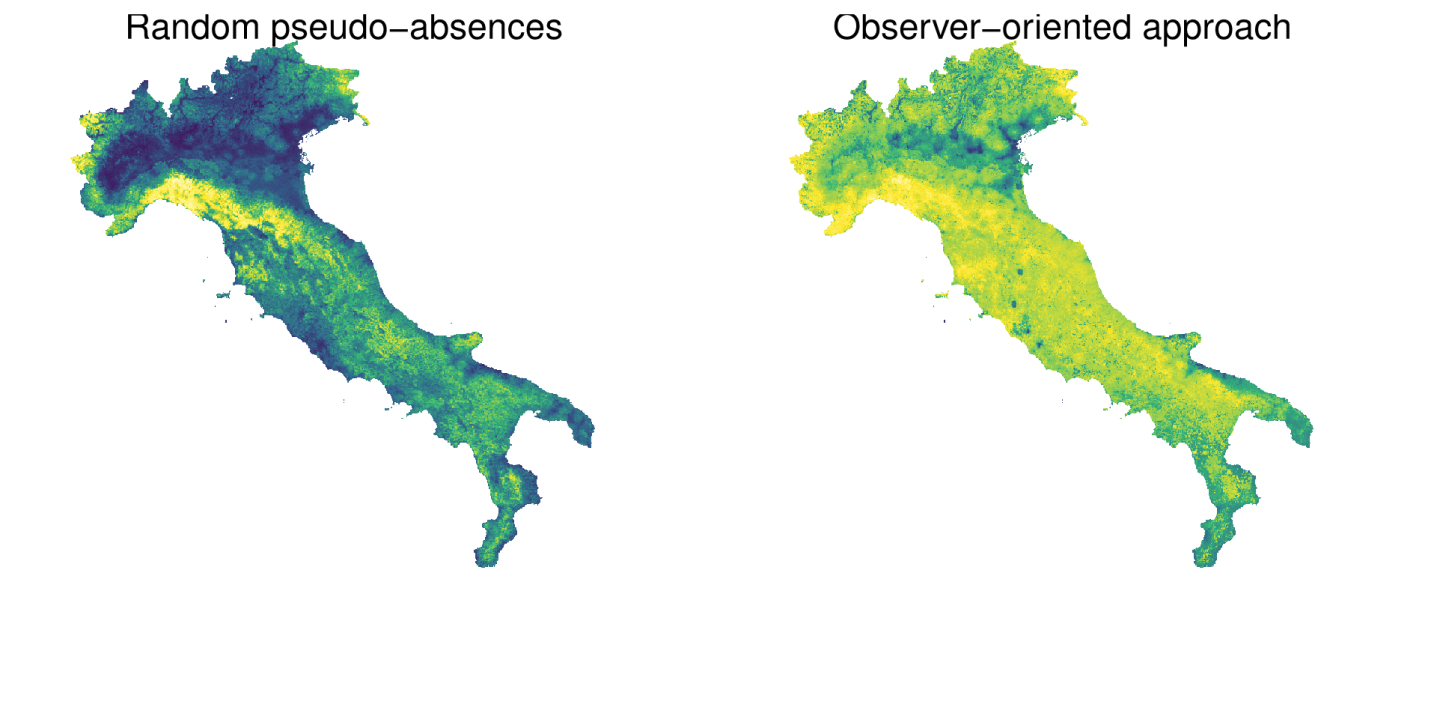


Figure S2. Resulting weighted ensemble predictions for the roe deer derived from nine different species distribution models carried out alternatively using random pseudo-absences (left) and observer-oriented’ approach (right). Blue-yellow scale indicates low-high probability of occurrence.


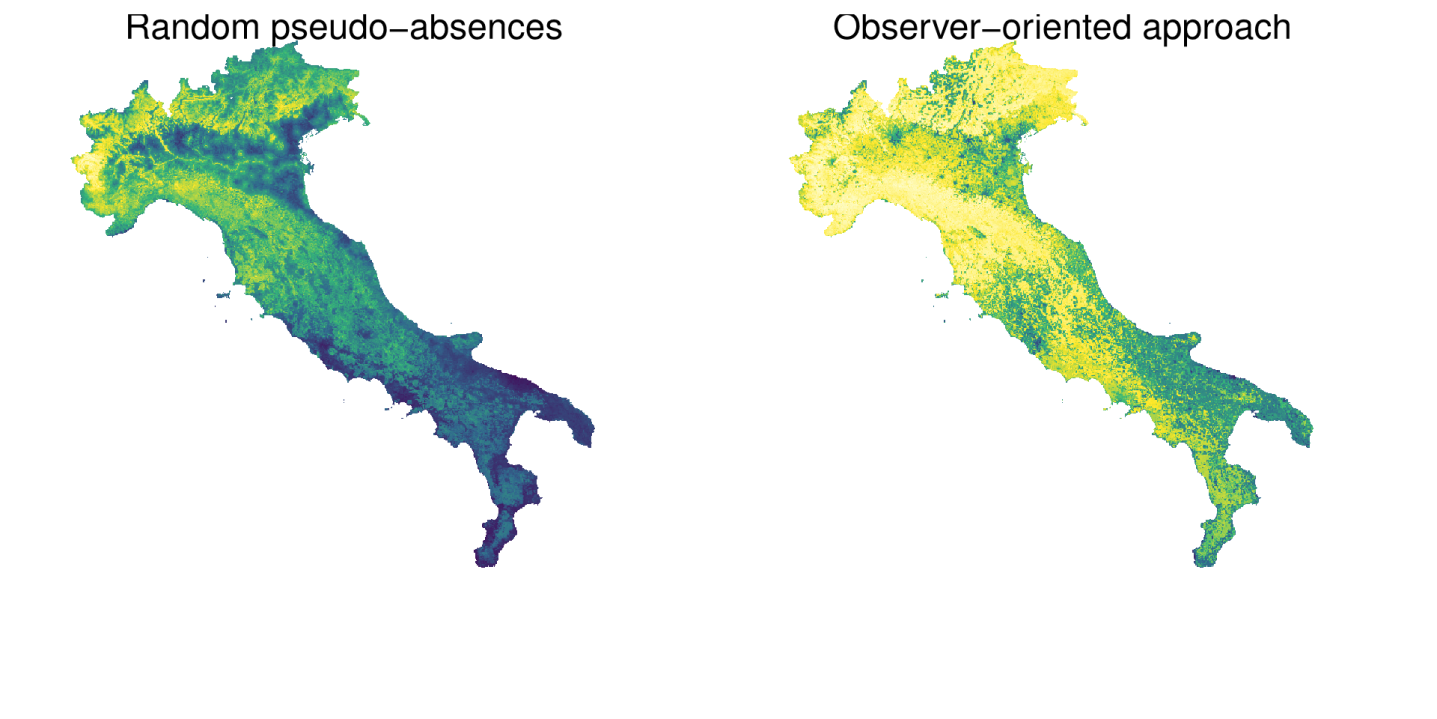


Figure S3. Resulting weighted ensemble predictions for the red deer derived from nine different species distribution models carried out alternatively using random pseudo-absences (left) and observer-oriented’ approach (right). Blue-yellow scale indicates low-high probability of occurrence.


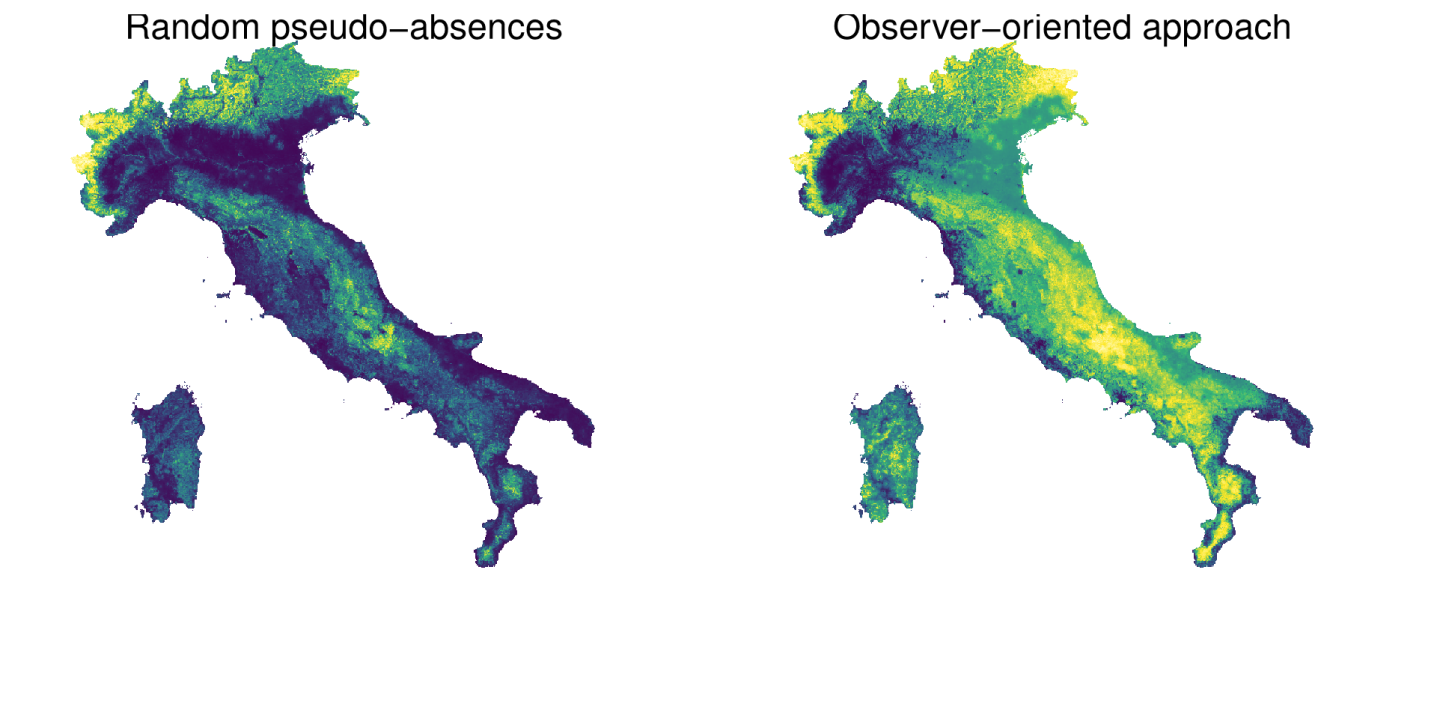


Figure S4. Resulting weighted ensemble predictions for the fallow deer derived from nine different species distribution models carried out alternatively using random pseudo-absences (left) and observer-oriented’ approach (right). Blue-yellow scale indicates low-high probability of occurrence.


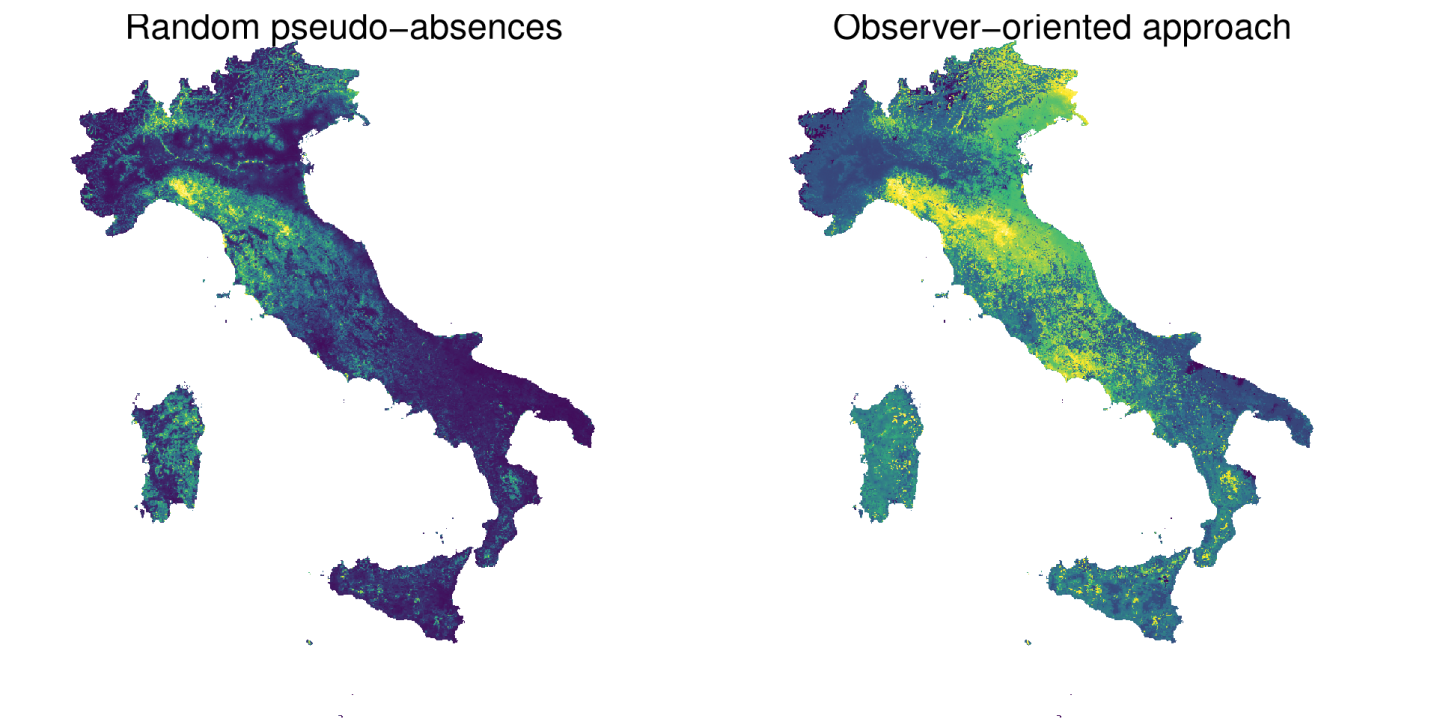


Figure S5. Resulting weighted ensemble predictions for the European hedgehog derived from nine different species distribution models carried out alternatively using random pseudo-absences (left) and observer-oriented’ approach (right). Blue-yellow scale indicates low-high probability of occurrence.


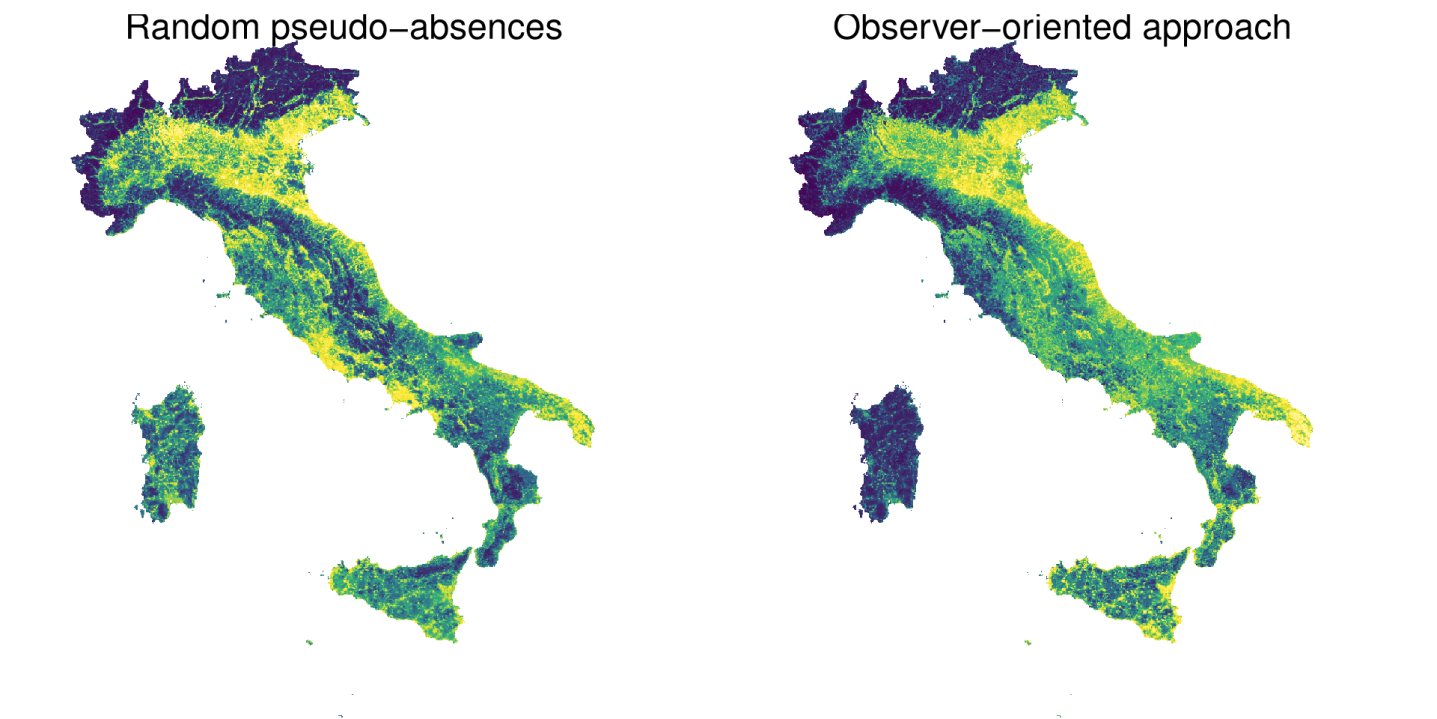


Figure S6. Resulting weighted ensemble predictions for the crested porcupine derived from nine different species distribution models carried out alternatively using random pseudo-absences (left) and observer-oriented’ approach (right). Blue-yellow scale indicates low-high probability of occurrence.


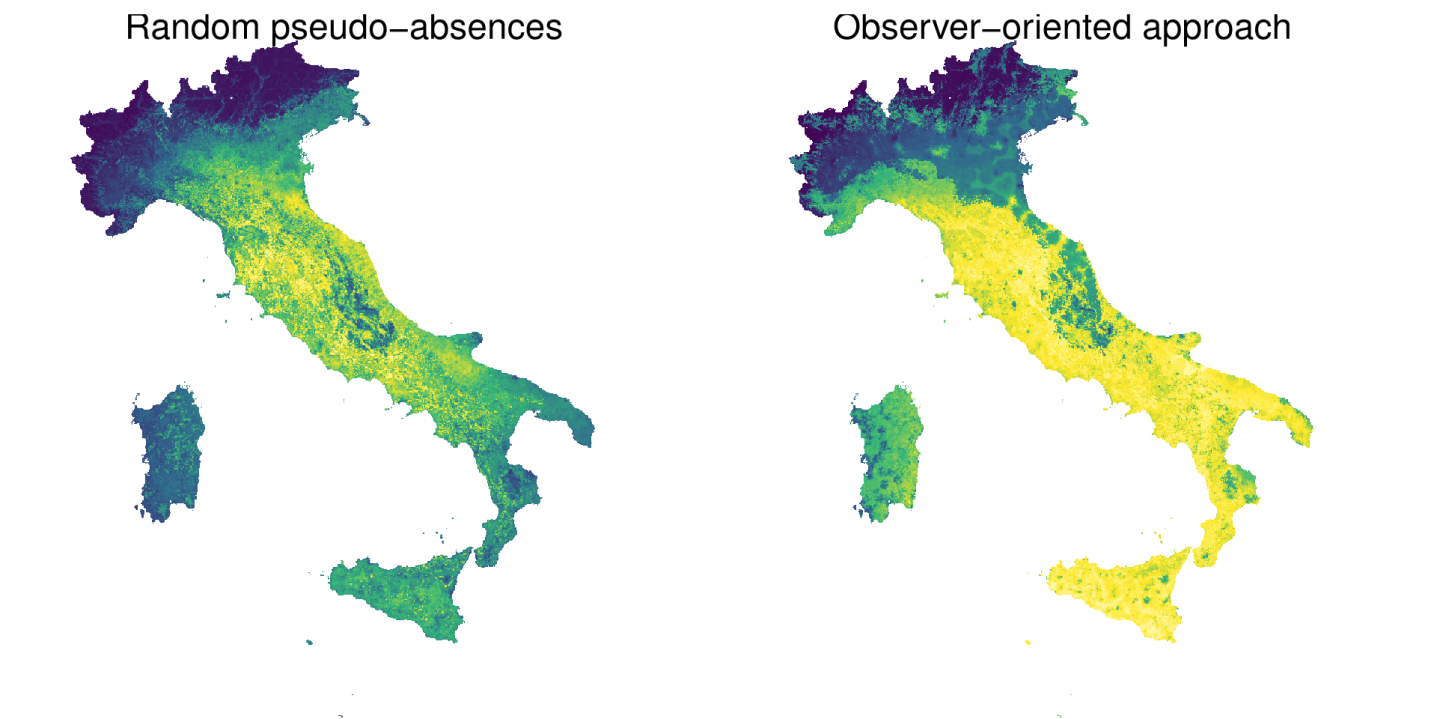


Figure S7. Resulting weighted ensemble predictions for the European brown hare derived from nine different species distribution models carried out alternatively using random pseudo-absences (left) and observer-oriented’ approach (right). Blue-yellow scale indicates low-high probability of occurrence.


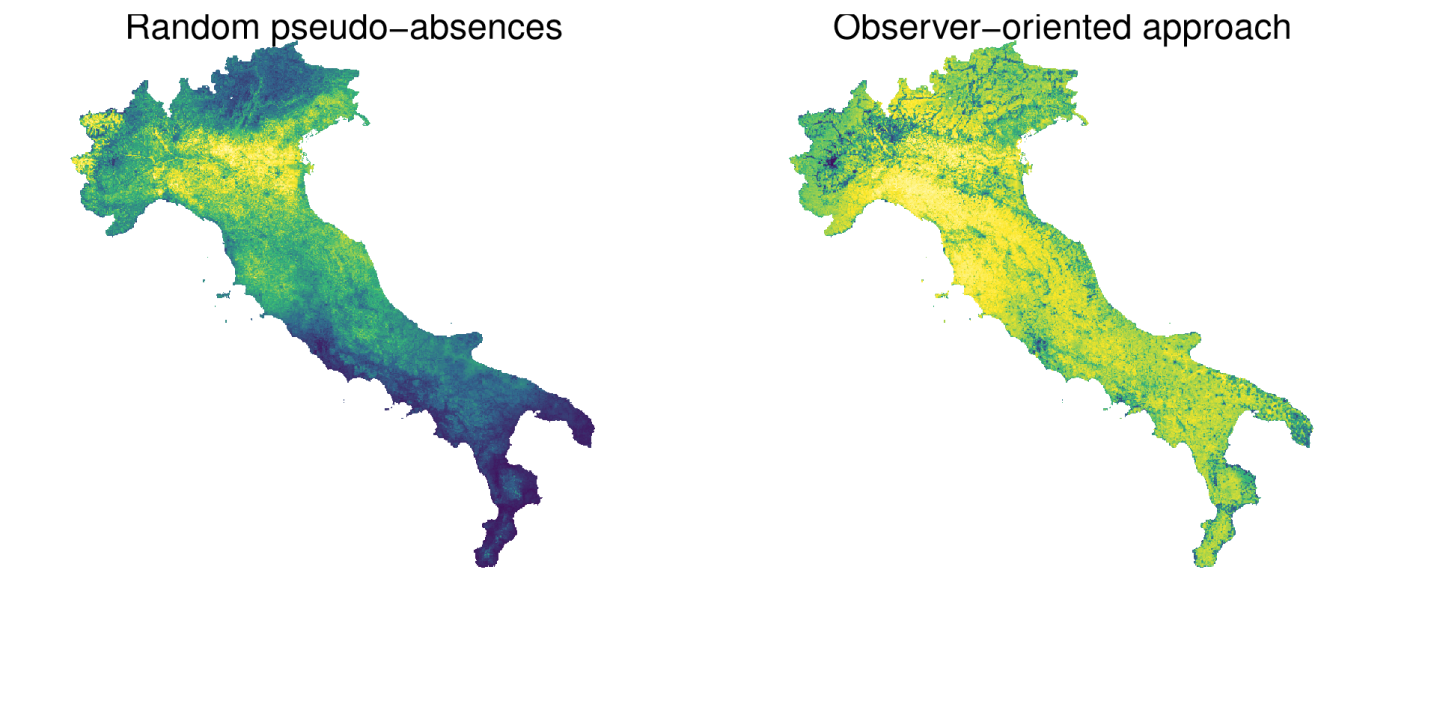


Figure S8. Resulting weighted ensemble predictions for the European badger derived from nine different species distribution models carried out alternatively using random pseudo-absences (left) and observer-oriented’ approach (right). Blue-yellow scale indicates low-high probability of occurrence.


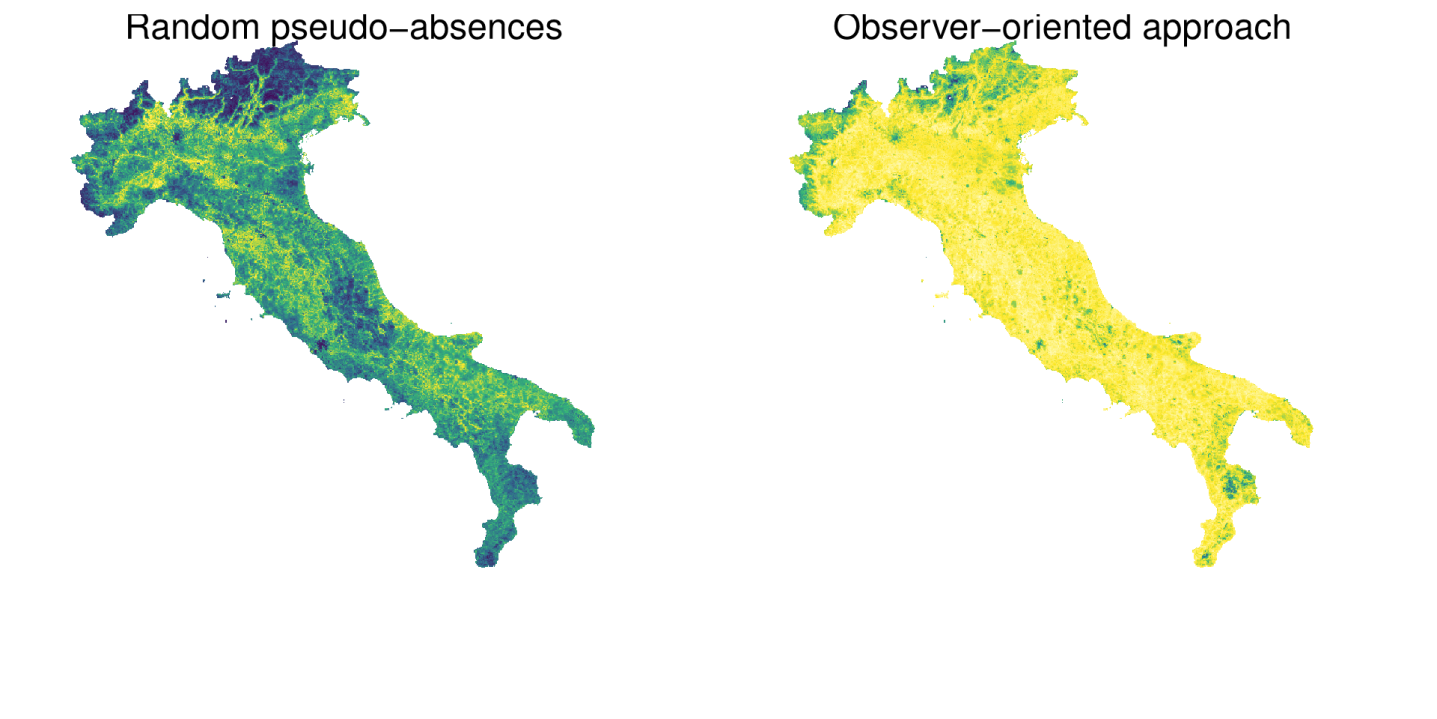


Figure S9. Resulting weighted ensemble predictions for the coypu derived from nine different species distribution models carried out alternatively using random pseudo-absences (left) and observer-oriented’ approach (right). Blue-yellow scale indicates low-high probability of occurrence.


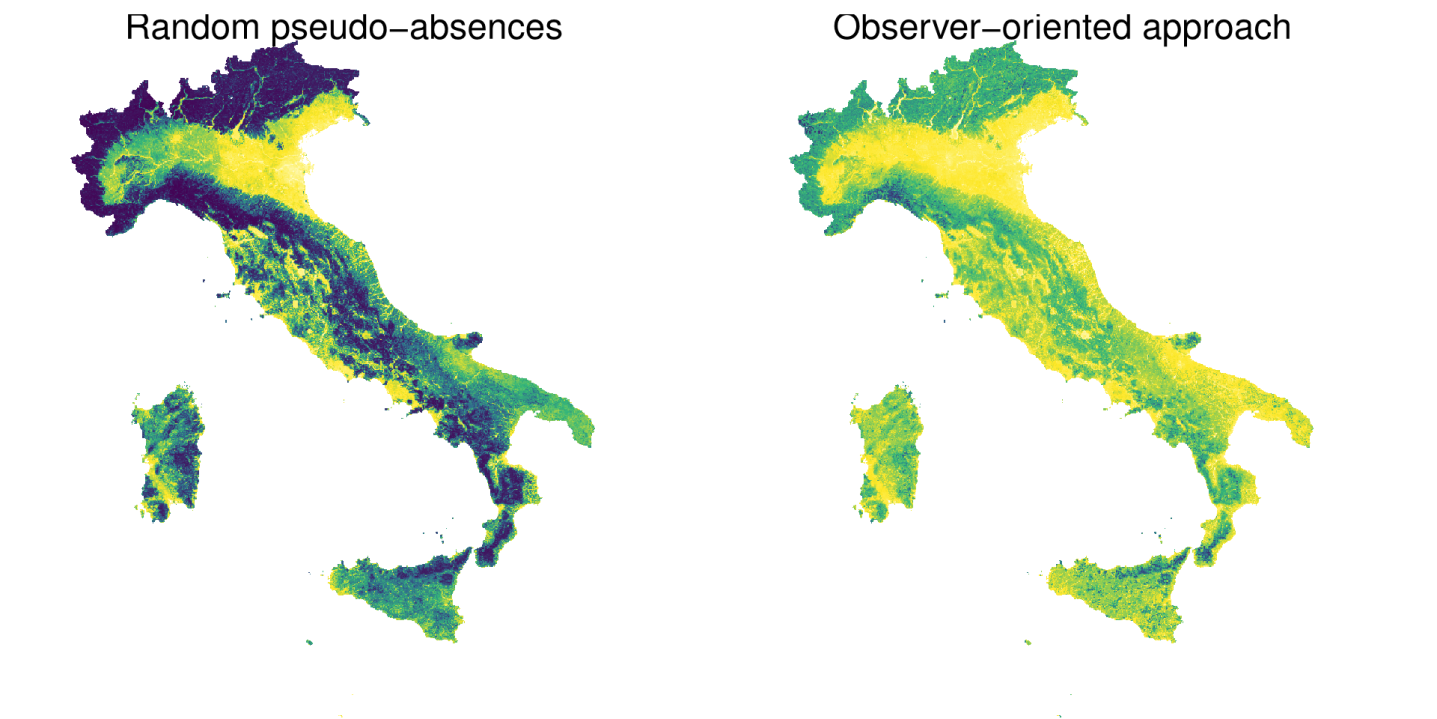


Figure S10. Resulting weighted ensemble predictions for the Northern chamois derived from nine different species distribution models carried out alternatively using random pseudo-absences (left) and observer-oriented’ approach (right). Blue-yellow scale indicates low-high probability of occurrence.


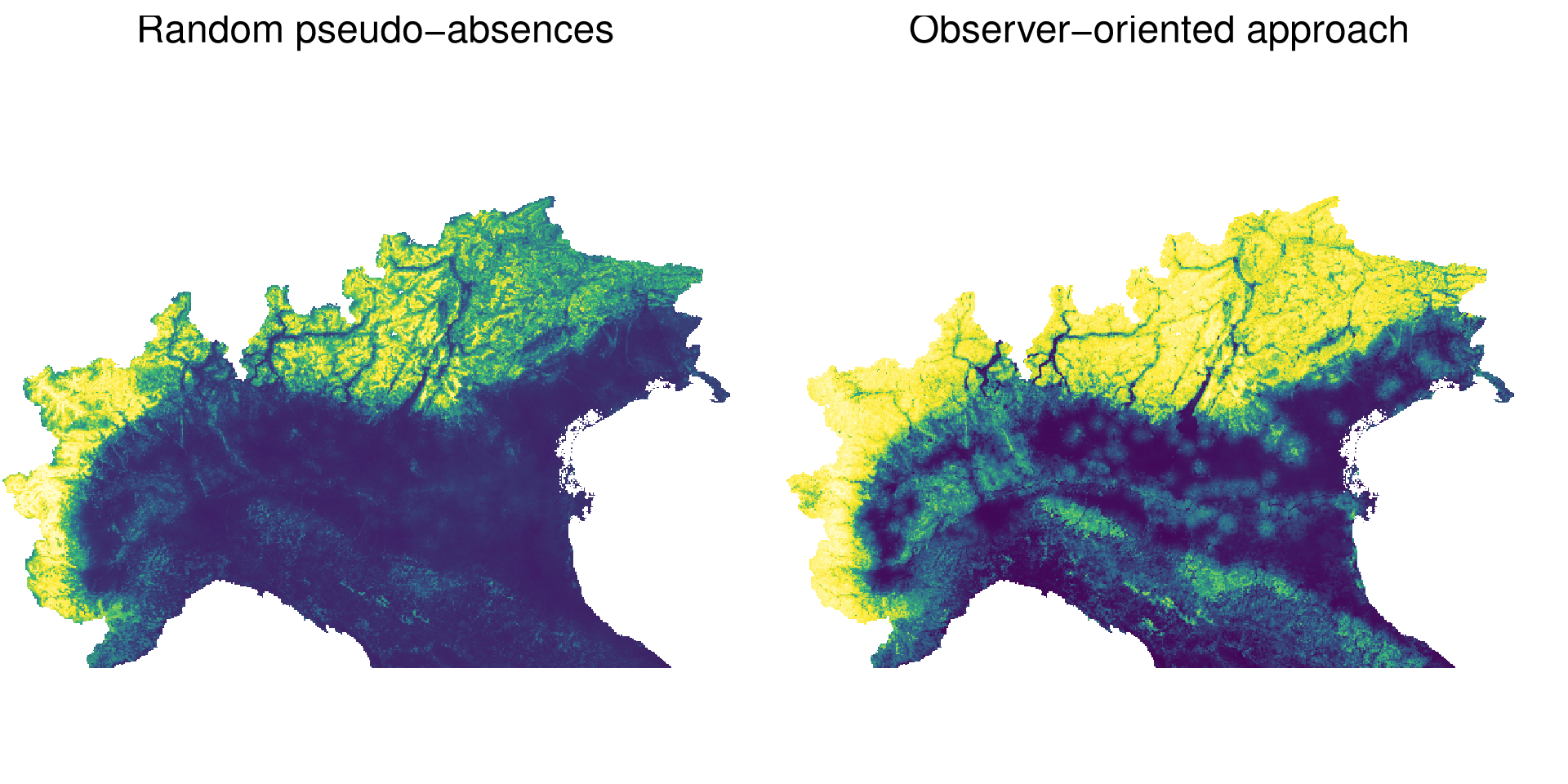


Figure S11. Resulting weighted ensemble predictions for the grey squirrel derived from nine different species distribution models carried out alternatively using random pseudo-absences (left) and observer-oriented’ approach (right). Blue-yellow scale indicates low-high probability of occurrence.


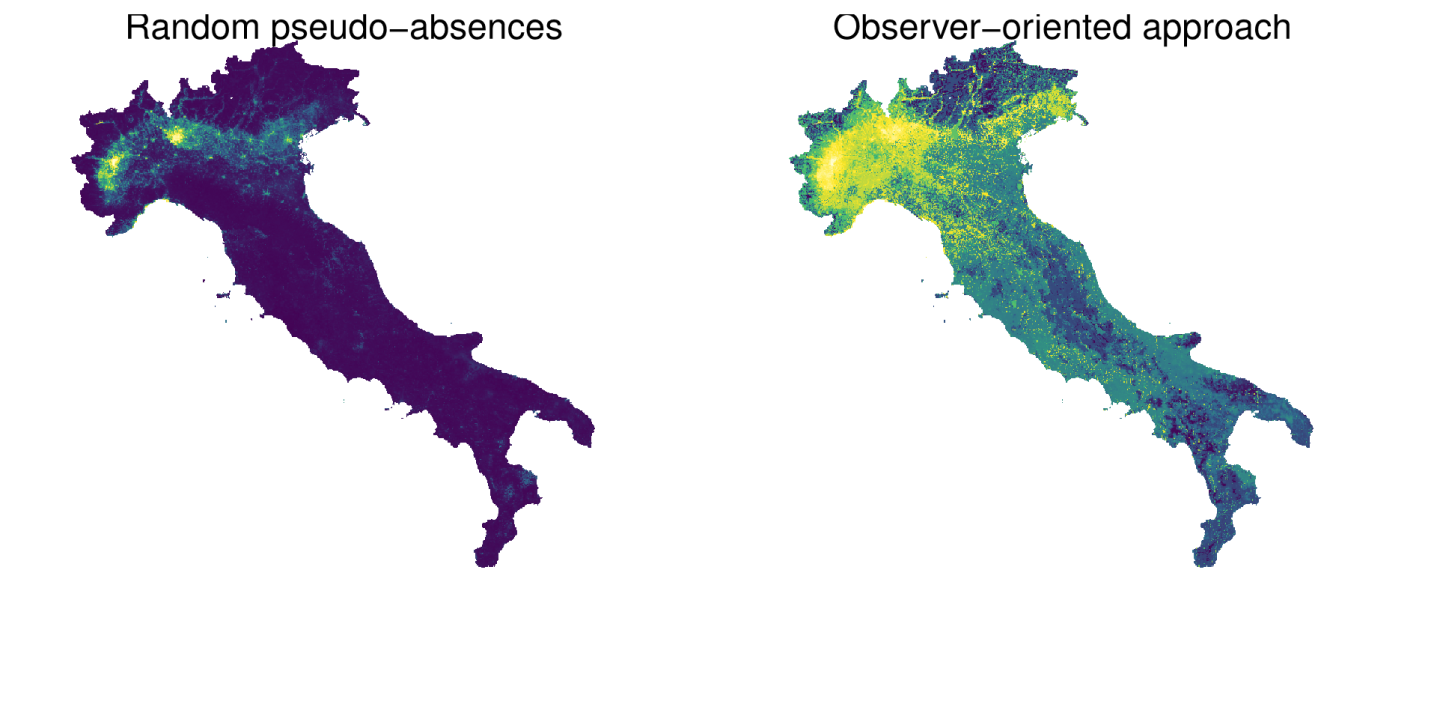


Figure S12. Resulting weighted ensemble predictions for the red squirrel derived from nine different species distribution models carried out alternatively using random pseudo-absences (left) and observer-oriented’ approach (right). Blue-yellow scale indicates low-high probability of occurrence.


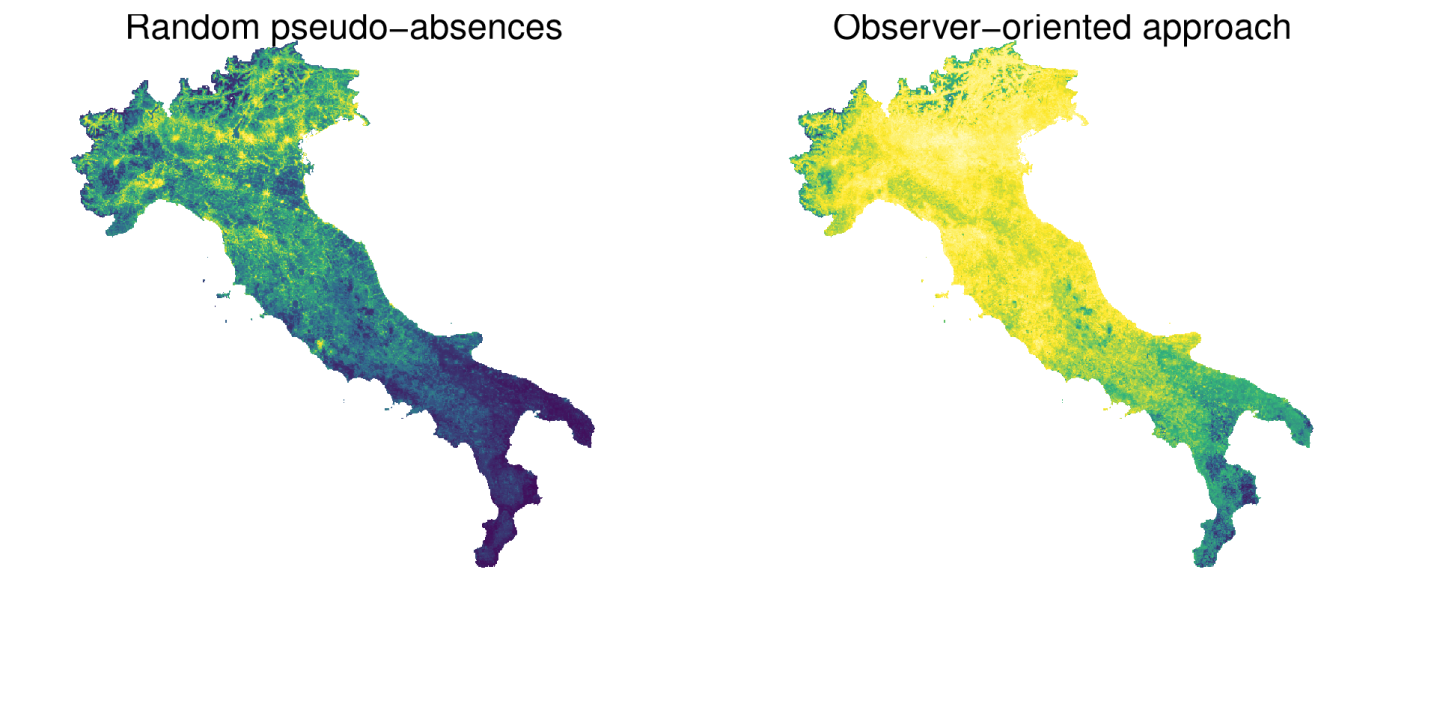


Figure S13. Resulting weighted ensemble predictions for the wild boar derived from nine different species distribution models carried out alternatively using random pseudo-absences (left) and observer-oriented’ approach (right). Blue-yellow scale indicates low-high probability of occurrence.


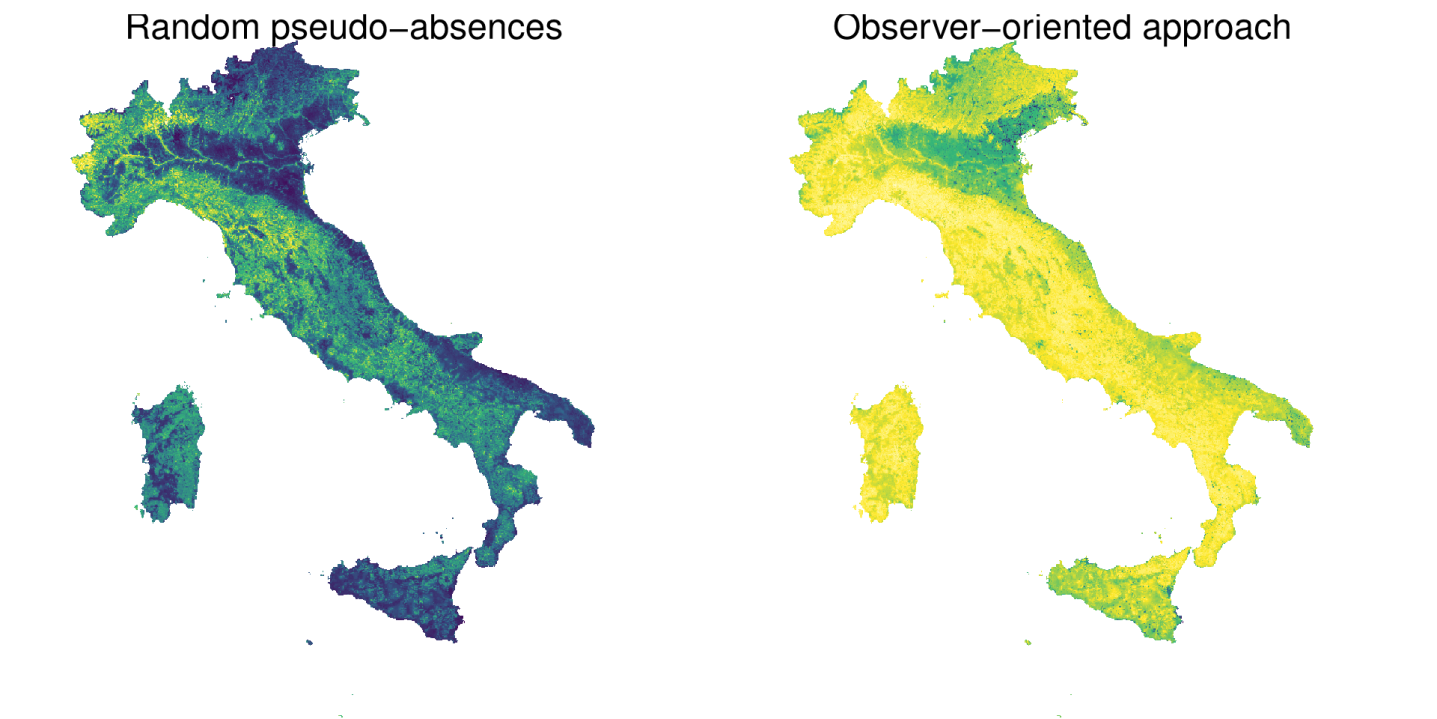


Figure S14. Resulting weighted ensemble predictions for the Eastern cottontail derived from nine different species distribution models carried out alternatively using random pseudo-absences (left) and observer-oriented’ approach (right). Blue-yellow scale indicates low-high probability of occurrence.


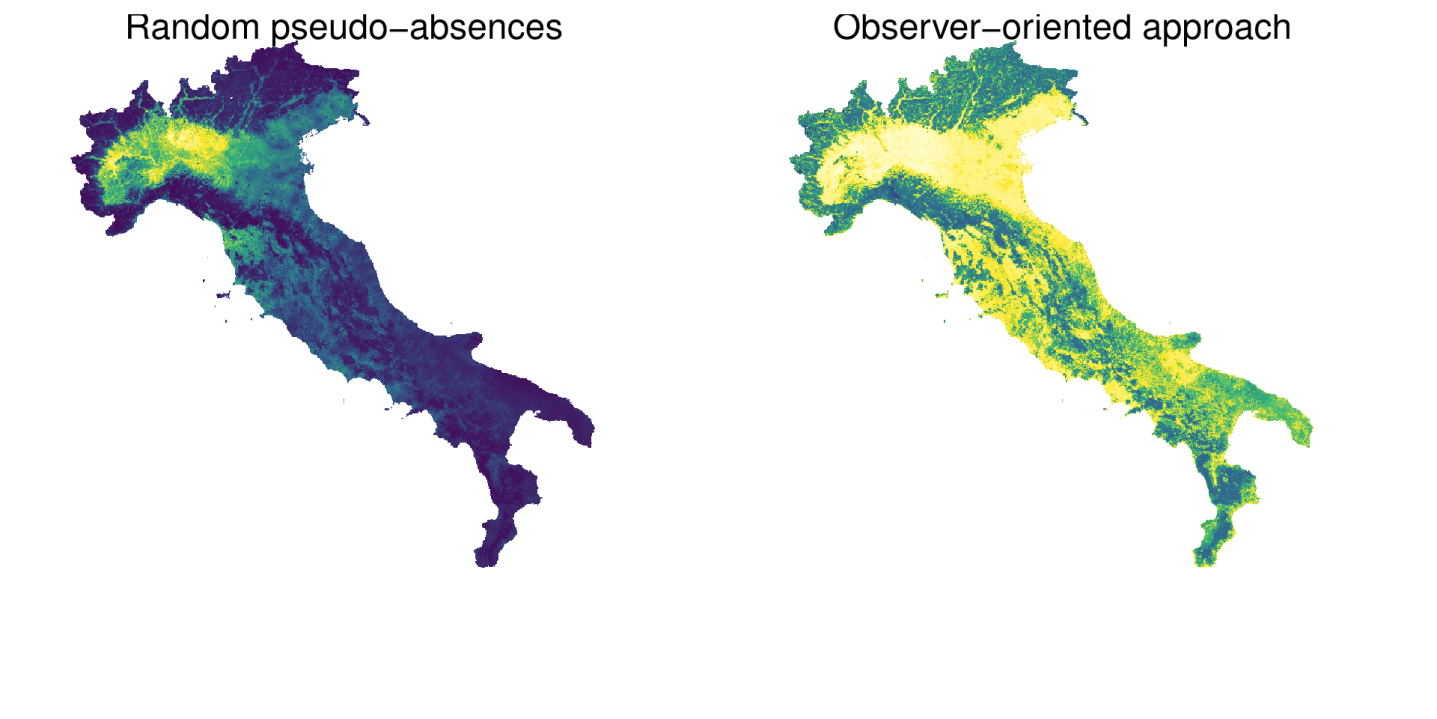


Figure S15. Resulting weighted ensemble predictions for the red fox derived from nine different species distribution models carried out alternatively using random pseudo-absences (left) and observer-oriented’ approach (right). Blue-yellow scale indicates low-high probability of occurrence.


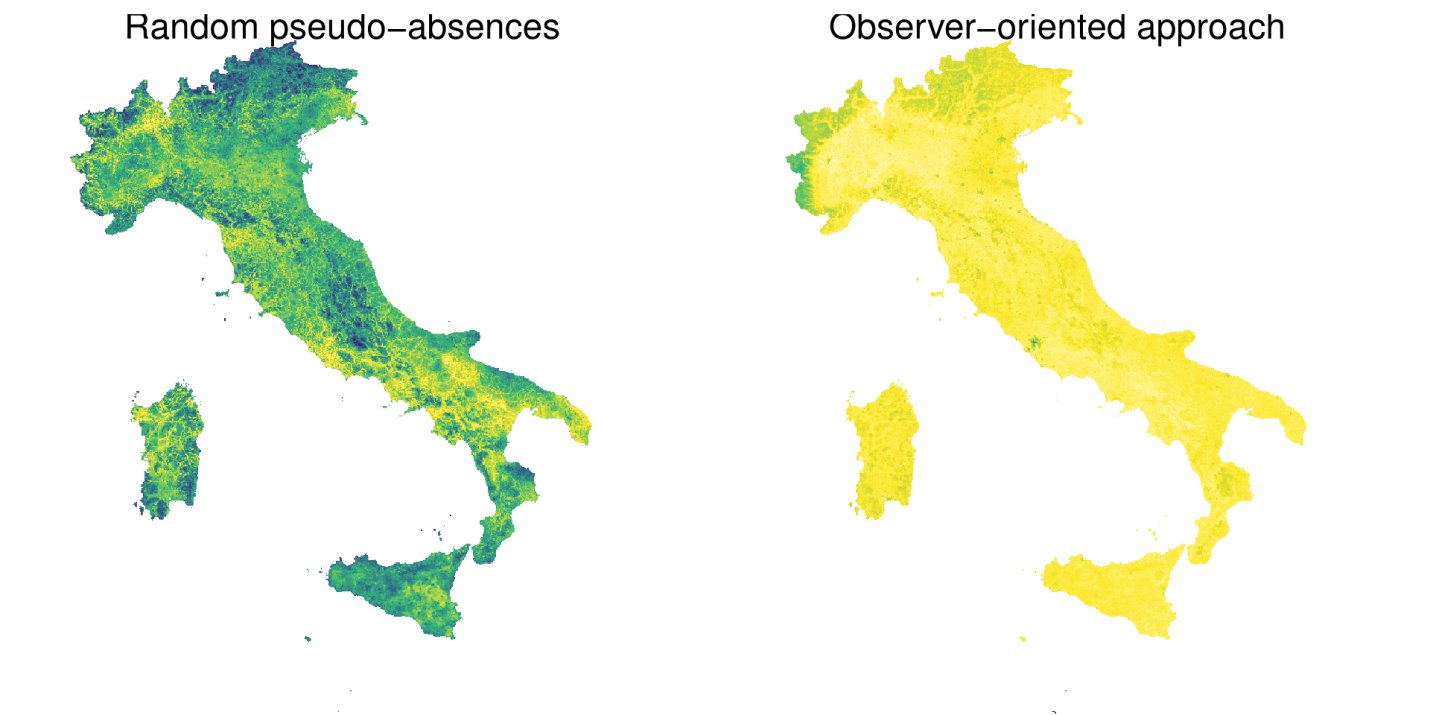

Supplement: Supplementary file 1 — Appendix [file ECE3-10-12104-s001.docx]
